# Spatio-Temporal Analysis of Urban Acoustic Environments with Binaural Psycho-Acoustical Considerations for IoT-Based Applications

**DOI:** 10.3390/s18030690

**Published:** 2018-02-26

**Authors:** Jaume Segura-Garcia, Juan Miguel Navarro-Ruiz, Juan J. Perez-Solano, Jose Montoya-Belmonte, Santiago Felici-Castell, Maximo Cobos, Ana M. Torres-Aranda

**Affiliations:** 1 Computer Science Department, Escola Tècnica Superior d’Enginyeria, Universitat de València, 46100 Burjassot, Spain; juan.j.perez@uv.es (J.J.P.S.); santiago.felici@uv.es (S.F.C.); maximo.cobos@uv.es (M.C.S.); 2 Grupo de Investigación en Telecomunicaciones Avanzadas (GRITA), Universidad Católica de Murcia (UCAM), 30107 Guadalupe, Spain; jmnavarro@ucam.edu (J.M.N.R), jmontoya3@alu.ucam.edu (J.M.B); 3 Departamento de Ingeniería Eléctrica, Electrónica, Automática y Comunicaciones, Escuela Politécnica de Cuenca (Universidad de Castilla-La Mancha), 16071 Cuenca, Spain; Ana.Torres@uclm.es

**Keywords:** acoustic environment, soundscape, WASN, psychoacoustics, IoT, spatial statistics

## Abstract

Sound pleasantness or annoyance perceived in urban soundscapes is a major concern in environmental acoustics. Binaural psychoacoustic parameters are helpful to describe generic acoustic environments, as it is stated within the ISO 12913 framework. In this paper, the application of a Wireless Acoustic Sensor Network (WASN) to evaluate the spatial distribution and the evolution of urban acoustic environments is described. Two experiments are presented using an indoor and an outdoor deployment of a WASN with several nodes using an Internet of Things (IoT) environment to collect audio data and calculate meaningful parameters such as the sound pressure level, binaural loudness and binaural sharpness. A chunk of audio is recorded in each node periodically with a microphone array and the binaural rendering is conducted by exploiting the estimated directional characteristics of the incoming sound by means of DOA estimation. Each node computes the parameters in a different location and sends the values to a cloud-based broker structure that allows spatial statistical analysis through Kriging techniques. A cross-validation analysis is also performed to confirm the usefulness of the proposed system.

## 1. Introduction

Noise has become a big problem in big cities. It has been shown that affects human behavior, health and even children’s cognition [1]. Different measurements and studies of this environmental factor have been performed in the past [2,3]. The normal way to do noise measurements is to collect noise samples with a sound level meter, however this technique has many drawbacks. On the one hand, only local measurements in a grid are taken and on the other, it is expensive due to the measuring equipment and personnel costs. Moreover, these studies have been performed observing objective parameters, the evaluation of the equivalent sound pressure level [4]. However, previous works have shown that the evaluation of psychoacoustic parameters, such as loudness and sharpness, fits better to the assessment of noise subjective annoyance [5,6]. Psychoacoustic research has been widely studied and standards for evaluating subjective annoyance and calculating psychoacoustic parameters have been created [7,8].

In 2014, the International Standardization Organization introduced the standard ISO 12913 about soundscape [9], following the recommendations of the COST action Soundscape [10]. The standard defines the soundscape as: acoustic environment as perceived or experienced and/or understood by a person or people, in context [9]. This context includes the relationships between person and activity and place, in space and time. The interaction between the different actors in the acoustic environment establish a dynamic context which is able to influence soundscape through different mechanisms as: (1) the auditory sensation, (2) the interpretation of auditory sensation, and (3) the responses to the acoustic environment. In this way, the soundscape differs from the acoustic environment (defined as: sound at the receiver from all sound sources as modified by the environment). In this work, the study is mainly oriented to the analysis of the acoustic environment, but as the evaluation is made in terms of the estimation of the psycho-acoustic annoyance (because it is not related to the direct subjective response of people) by using the Zwicker’s model. Here there is no specific qualification of the sound by subjective perception, but the description of the environment with a simplified version of the Zwicker’s model is used. This can be related to the subjective response, but this will be done in a later study. Other aspects that tackle this study are related to the development of an infrastructure to evaluate the acoustic environment in terms of binaural psycho-acoustic parameters and also its spatial and temporal analysis.

In the context of modern urban planning and smart cities, IoT systems are receiving increasing attention due to their ability to monitor different problematic areas within a population center [11,12]. These problems are mostly related to the environment, but also to e-Health, Intelligent Transportation Systems (ITS) and other emerging technological areas with high demands [13,14,15].

This work explains the implementation of an IoT-based system oriented to gather binaural psycho-acoustic information from indoor and outdoor environments. As the annoyance model proposed uses only loudness and sharpness as the main parameters, some simplifications have been made. In order to explain the performance of this parameters in the considered environments, spatial and temporal analysis have been done in the measurements to check the validity of the spatial statistical model proposed using kriging.

The paper is organised as follows: in Section 2, a brief state-of-the-art is sketched. In Section 3, the theoretical and practical methodology used to deploy the experiments, both in indoor and outdoor environments, with spatial statistical analysis (using kriging method) are described, discussing also hardware, software and signal processing considerations. Also in the previous section, the procedure to obtain the binaural loudness and sharpness is described. In Section 4, the results are presented and discussed. Finally, Section 5 summarizes the main conclusions of this work.

## 2. Related Works

Different authors have implemented different monitoring systems for environmental noise and have evaluated the related annoyance. The use of WASNs for noise monitoring was the main focus of the works in [16,17]. The authors used a WASN with Tmote-Sky motes [18] and Tmote Invent (TmI), for traffic noise monitoring using the equivalent level ($L_{eq,T}$) and counting the number and type of vehicles. In that deployment, the sampling frequency was set at 8 kHz. As an important conclusion, the authors stated that Tmote-Sky had excessive self-noise making it inappropiate for acquiring sound measurements, while, TmI offered good audio features. In that research, no specific procedure for calibration was provided. In [19,20], the authors deployed a WASN in Ostrobothnia (Finland). In these references, the authors evaluated different tests to assess the noise impact. They measured the $L_{eq,T}$ with T=125 ms using a sampling frequency of 33 kHz, with 14 calibrated nodes (MicaZ from Crossbow -now MEMSIC- with an ad-hoc acquisition circuit to allow a dynamic range of 60 dB), and synchronised during 96 h, they obtained good results. Other works such as [21,22,23] used mobile phones for noise pollution monitoring. Although the authors obtained interesting results, in this paper there is a lack of information about the recording conditions which means less accuracy in the noise measurements. When evaluating noise parameters, the location of the measuring devices should follow specific rules [24].

In the previous works [16,17,18,19,20,21,22,23], the measurements focused on the $L_{eq,T}$ or its A-weighted version (ITU-R 468), $L_{eqA,T}$. The calculation of these measurements was made by applying a frequency-selective filter that considers the frequency range around 3–6 kHz, where the human ear is most sensitive.

From the previous references, we can see similar works more oriented to the soundscape description. In [25,26], the referred authors focus the description of the soundscape in the evaluation of noise levels and psycho-acoustic parameters from recordings gathered in walk-around. However, none of them include an analysis based on binaural psycho-acoustic annoyance, which probably should be more accurate from the psycho-acoustic point of view in a human being.

In the literature, the evaluation of Psycho-acoustic Annoyance (PA) is mainly based on the work by Zwicker & Fastl [27]. Different authors have tried to focus this research taking into account the subjective perception combined with specific psychoacoustic parameters [28]. Loudness is an important factor affecting the perceived psychoacoustic annoyance of a sound [27,29]. This parameter is defined as the subjective intensity of sound, which qualifies sounds ranging from quiet to loud. It is mainly related to the perceived amplitude of a sound. There are some models to evaluate this parameter which deliver a numerical estimation of the loudness level based on some objective characteristics of the sound. The best known models are Zwicker’s [27] and Moore’s [30], which are standardised in the 2 parts of ISO 532 [8]. They are based on a monaural stimulus and pass through different filtering stages which are similar to the ear natural filters and simulates the hearing system. Recently, new research in psychoacoustics has shown that monaural loudness is not enough to assess the acoustic loudness and different models for binaural loudness were developed [28,31]. The implementation of the Zwicker’s model [32] allowed a computing perspective for loudness monitoring. In a previous work [33], the authors developed a binaural loudness monitoring system by using the Zwicker’s model and using binaural synthesis by means of a combination of Head-Related Transfer Functions (HRTFs) and microphone array processing.

In [34], the authors evaluated the environmental noise with a mobile application and display the results in a map using the kriging method. In [35], the authors measured 5 minutes sound pressure level (SPL) values with a WASN and also evaluated spatial cross-validation using the kriging method in a small city in Spain. In [36], the authors implemented a system oriented to compute the psychoacoustic parameters in the server side from recorded audio chunks. In [33], the authors implemented a edge-computing system by using different Raspberry Pi 3 (Rpi3) nodes in order to perform an evaluation of performance when computing the binaural loudness directly on the Rpi3 nodes.

In this paper, an improvement of the previous work [33] with the binaural loudness is done by incorporating into the model the computation of the binaural sharpness. Moreover, a simplified version of the Zwicker’s psycho-acoustic annoyance model is evaluated (assuming specific conditions), mainly to assess the spatial distribution of the subjective annoyance in an indoor and outdoor environments.

## 3. Methodology

The implementation of a WASN to evaluate a simplified version of the psycho-acoustic annoyance based on an IoT framework considers different steps: (a) the hardware and software implementation of the nodes for the computation of psycho-acoustic parameters, (b) the IoT framework configuration and (c) the cross-validation and spatial statistical analysis.

### 3.1. Binaural Sharpness

From the Zwicker’s model, the sharpness [37] defined as the centroid of the spectrum can be calculated [38] by using the Equation (1)

(1) $$S=0.11\cdot \frac{\int_{0}^{24Bark}L^{\prime }\left(z\right)\cdot g^{\prime }\left(z\right)\cdot z\cdot dz}{\int_{0}^{24Bark}L^{\prime }\left(z\right)dz}$$

where for z<14, $g^{\prime }\left(z\right)=1$ and for z≥14, $g^{\prime }\left(z\right)=0.00012\cdot z^{4}-0.0056\cdot z^{3}+0.1\cdot z^{2}-0.81\cdot z+3.51$. $L^{\prime }\left(z\right)$ is the specific loudness, which depends on the frequency in terms of Bark (i.e., *z*).

In [33], the procedure to obtain the binaural loudness is described. In the present work, the binaural sharpness calculation is also implemented in order to evaluate a simplified version of the psycho-acoustic annoyance. As the specific loudness is obtained for each 1/3-frequency band, left and right sharpness is calculated using the weighting function $g^{\prime }\left(z\right)$ described in Equation (1) to compute the value of the sharpness in each channel. In order to compute the binaural sharpness, the same proportion value as in binaural loudness calculation (see Equation (2)) is applied [28].

(2) $$BS=\frac{3}{4}\cdot \left(S_{l}+S_{r}\right)$$

### 3.2. Simplifications for the Zwicker’s Model

In this work, a simplified implementation of the Zwicker’s annoyance model is proposed. Assuming, in this case, locations where the environmental noise have few tonal components that provokes low inter-modulation in the environmental sound were selected. Here, the model could be simplified by reducing Fluctuation Strength (F) and Roughness (R) components and making the term $w_{FR}\approx 0$ in the Zwicker’s annoyance model [27]. With this simplification the equation for the PA is presented in Equation (3)

(3) $$PA=L_{5}\cdot \left(1+w_{S}\right)$$

where $L_{5}$ is the percentile 5 for the loudness, and $w_{S}$ is $\left(S-1.75\right)\cdot 0.25\log_{10}\left(L_{5}+10\right)$ if sharpness S>1.75 and 0 if sharpness S≤1.75.

Here this simplification is needed, because at least at this moment the computation of binaural loudness and sharpness takes a long time (5–6 s for a 1 s of recorded audio) with the Rpi3 nodes. The use of more parameters in the model would collapse the computing capacity of the node for monitoring uses.

The binaural psycho-acoustic annoyance (BPA) is introduced here like the PA but using the binaural parameters (i.e., binaural loudness and binaural sharpness) to compute this binaural annoyance.

### 3.3. Hardware and Software Implementation

The sensing and computing device is composed of two subsystems: the acquisition subsystem and the processing core. The processing core is based on a Raspberry Pi [39] and the acquisition subsystem is composed by an array of four microphones of a Sony PlayStation3 Eye camera (PS3-Eye). The PS3-Eye microphone array works to process each channel using 16-bit depth and a sampling rate of 16 kHz, with a signal-to-noise ratio (SNR) of 90 dB and a power consumption of 500 mAh. Different works have used this device, mainly due to its camera, e.g., for eye tracking systems [40] or to improve the precision of multi-touch displays [41], but also for sound source mapping [42]. In this work, the principal device used is the microphone array for the audio capture of the acoustic sensor device. The distance between the outermost microphones is 62 mm, and the two middle microphones have reversed polarity. The device was located in two different environments (one indoor and one outdoor).

The core of the processing part of the node is a Rpi3 Model B, that it is used in the acquisition and publishing stages. The technical characteristics of the Rpi3 include a 1.2 GHz 64-bit quad-core ARMv8 CPU, 1 GB RAM, 40 GPIO pins, 4 USB ports, a full HDMI port, an 10/100MB Ethernet port and integrated 802.11n Wireless LAN, and also Bluetooth Low Energy (BLE). Also, the USB ports and the GPIO pins are a good solution, providing the Rpi with the possibility to have a range of peripherals available, such as WiFi antennas, ZigBee modules, microphones, cameras and connections with other devices, e.g., Arduino. A Power-Over-Ethernet connection [43] was chosen to power the board, in which an Ethernet cable provide both electrical power and data connection to the Rpi3.

**Figure 1:**
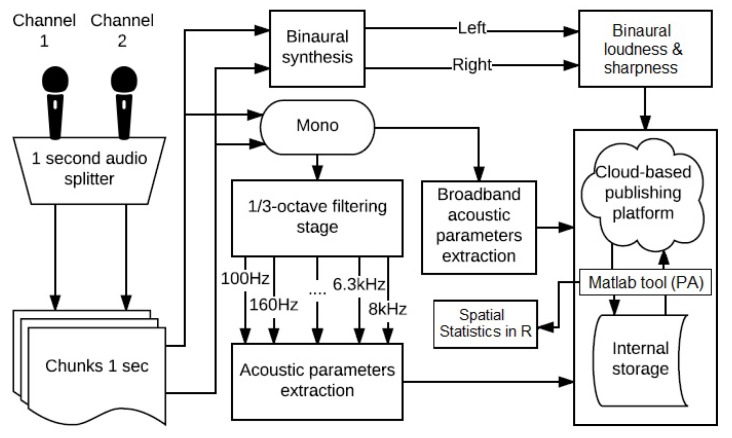
Diagram of the stages of the algorithm: acquisition, loudness and sharpness evaluation, binaural processing, sound pressure level calculation, and publishing, storing and processing (PA computation and spatial statistic processing) of the results.

The design of the device was made according to the model shown in Figure 1 [33]. In the software section, the Rpi3 platform has a Linux-based OS used for the programming in a high-level programming language, such as Python. The main tasks of the developed algorithm are: acquisition and windowing of the audio signal, binaural synthesis, loudness and sharpness calculation, binaural psycho-acoustic parameters calculation, monoaural signal creation, third-octave band filtering stage, sound pressure level calculation, storage, publishing and processing of the results. Also, a copy of the different parameters evaluated is stored in the internal memory of the Rpi3 sensing device.

First, the acquisition stage is carried out, recording the audio from the microphone array in chunks of 1 second. Direction-Of-Arrival (DOA) estimates for a set of frequency bins are obtained by processing the audio input from the two central microphones in the Short-Term Fourier Transform (STFT) domain [33,44,45]. Then, in the following stage, the binaural synthesis is performed to create a signal that imitates the human hearing system response by using HRTFs and the DOA information resulting from the previous stage. Next, the binaural loudness and binaural sharpness are calculated from the binaural signal synthesised. Finally, the results are uploaded to a Cloud-based service (namely ThinkSpeak) for storing and publishing data. This service allows data processing, using the ThinkSpeak API, allowing the connection with Matlab. This connection allows to compute the PA, based on the approximation explained in Section 3.1 by Equation (3) and finally, the results are analysed in RStudio to investigate the temporal and spatial correlation between the different nodes. Then, the spatial statistics of the environment is analysed using the kriging method [46,47], in order to assess the most annoying areas. This analysis is made by determining the interpolated PA in different locations between the nodes and considering the error of this method. Finally, the evaluation is conducted following a leave-one-out cross-validation approach with a linear model technique and analyzing the Root Mean Squared Error (RMSE).

At the same time, before the binaural synthesis stage, the monophonic channels are converted into a monaural signal to extract acoustic parameters of the full spectrum Sound Pressure Level (SPL) and from a third octave band A-filtering stage SPL (SPL(A)). In reference [30], the authors describe in detail the calibration procedure of the nodes for the measurement of the SPL and the binaural loudness and sharpness. Finally, these results are sent to the cloud and to the internal memory too. Also, the correlation between temporal evolution of these parameters and the psycho-acoustic one’s is determined.

## 4. Results and Discussion

In this section, the WASN performance is evaluated carrying out two experiments in an indoor and outdoor environment. Moreover, the results of these experiments are presented and analyzed.

**Figure 2:**
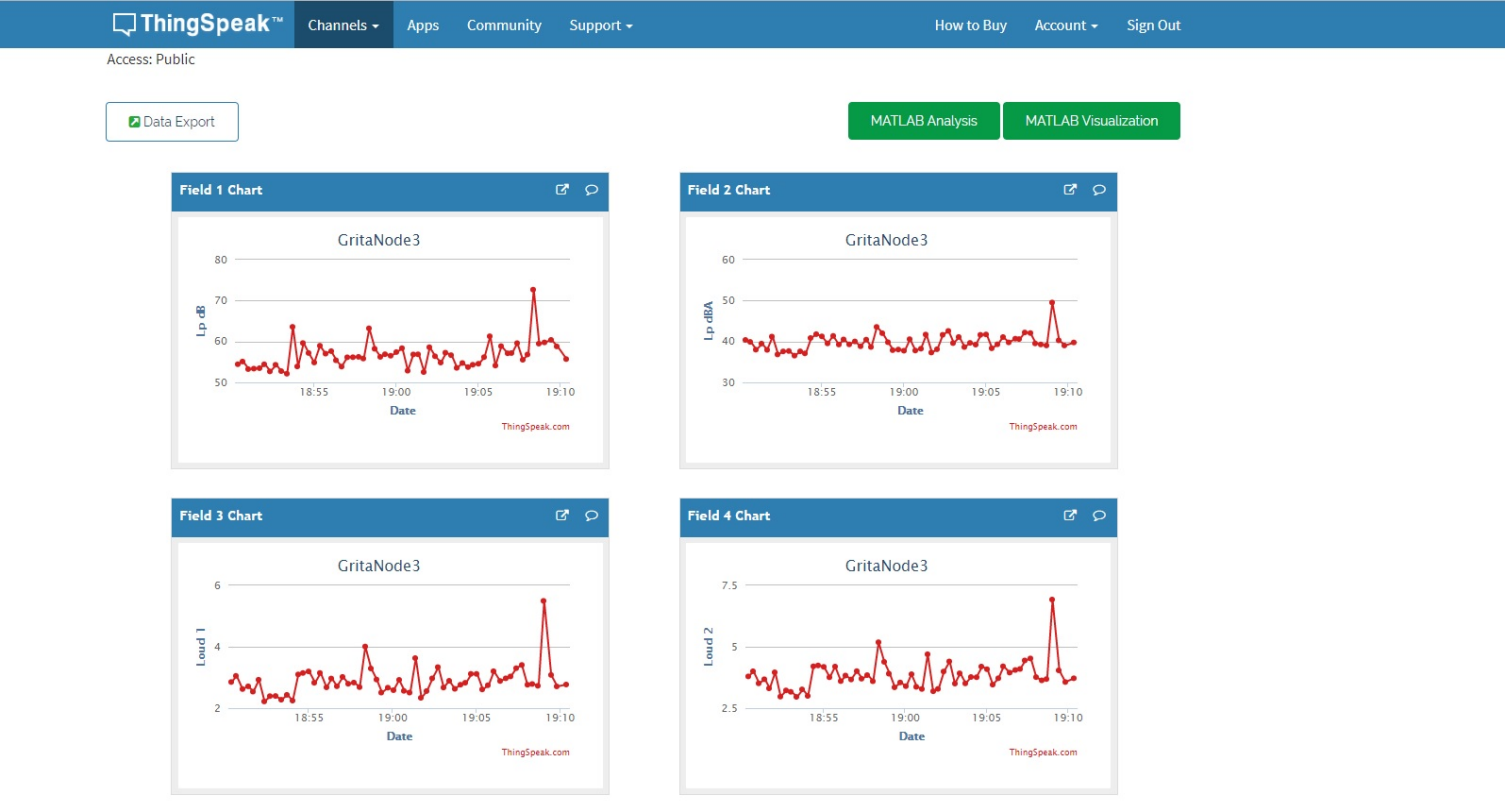
Channel configuration detail in the ThingSpeak platform.

The experiments planned for this work consider an indoor and an outdoor environment. The indoor environment corresponds to the location of 4 nodes in different offices of the Computer Science Dpt at the ETSE in the University of Valencia and the outdoor environment corresponds to the location of different nodes in the façades of houses in a neighborhood from Murcia city close to a highway. The system performance is evaluated and the results are presented and analysed. Figure 2 shows the ThingSpeak configuration for the data channels in the indoor environment, collecting SPL, channels left and right for loudness (L), binaural loudness (BL), channels left and right for sharpness (S), and binaural sharpness (BS).

### 4.1. Indoor Environment

**Figure 3:**
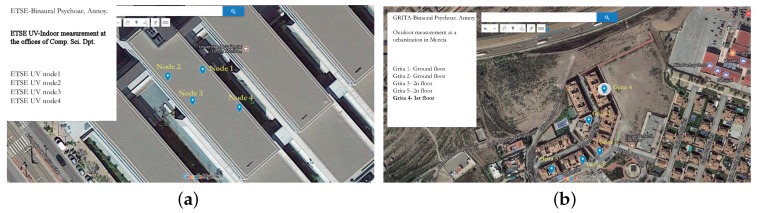
Location of the measurement points for the indoor at ETSE (University of Valencia) (**a**) and outdoor measurements at an urbanization near Murcia (**b**).

The different nodes considered in this experiment were placed at different offices in the third floor of the first block in the ETSE. Here two offices (one with a professor and fluent activity of students during few hours and other with only a professor working), the secretary office of the department and a laboratory with one student working during several hours were selected. Although the activity was different in each location, it was not too high, therefore the noise levels and the computed psycho-acoustic annoyance had low variability. All the nodes were connected to Internet and every node was linked to a different channel in the ThingSpeak platform during 8 h. Figure 3a shows the location of the four nodes from the upper part of the building.

Table 1 shows the statistical values (mean and standard deviation) for each node for the indoor measurements. Table 2 shows the correlation matrix of binaural loudness, binaural sharpness and SPL.

### 4.2. Outdoor Environment Measurement

The outdoor environment corresponds to an urbanization near a highway. Therefore, the traffic noise is considerable. The measurement points were located in two different lines of buildings. The buildings in the second line (far away from the highway) are more protected from the traffic noise, but these buildings are near a pedestrian area where some children were playing during few hours. In this two different areas, the activity is also the criteria selected. In this location, five nodes were placed in different façades of the urbanization houses. Nodes 1 and 2 were located in the ground floor, nodes 3 and 4 were located in the second floor and node 5 was placed in the first floor. All of them in the façade of the corresponding house. Figure 3b shows the location of these nodes.

Table 3 shows the statistical values (mean and standard deviation) for each node for the outdoor measurements. Table 4 shows the correlation matrix of binaural loudness, binaural sharpness and SPL.

As shown in Table 2 and Table 4, correlation of the temporal series of the different considered parameters (i.e., SPL, binaural loudness and binaural sharpness) show clear differences. This fact is related to the different nature of the parameters considered. In general, SPL is more related to loudness, but sharpness differs, because it depends mainly on the centroid of the spectral content of the signals in each environment.

### 4.3. Modelling and Cross-Validation of the Measurement Sets

**Figure 4:**
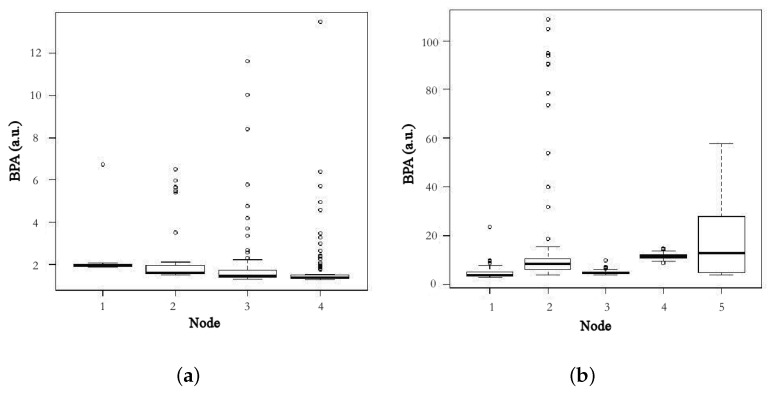
Box diagram of the BPA indoor (**a**) and outdoor values (**b**).

Figure 4a,b show the results of the descriptive analysis of the BPA in every location in the indoor and the outdoor environment.

In order to perform a validation of the values and the number of selected points for the linear modeling of the set of measured values, a Leave-One-Out-Cross-Validation algorithm in R-Studio software package (with the “Linear Model” method) was used for the whole set of time-averaged measurements, corresponding to the spatial locations. In Table 5 the results of this test are shown in order to select the nodes in the indoor environment. A linear model is generated to predict the % established in the first column (%test/%training) with the mean value of the BPA in every location. In this table, *Rsquared* is the Pearson’s squared correlation coefficient and *MAE* is the maximum absolute error. *RMSE* is the minimum of the list of results obtained with this algorithm. According to this table, the selection of a validation set with 60% of the nodes is enough for our network. Table 6 shows the results of the cross-correlation test to predict the temporal values of BPA in the indoor environment. In addition, a linear model is generated to predict the values of BPA in the test percentage established in the first column (as in the previous case). Here, the whole temporal series was used in each location and from these results we can predict with high accuracy the test values training the model with the 50% of the measurements (as shown the RMSE and MAE values).

In Table 7, the results of the test to select the nodes in the outdoor environment are presented. This prediction is made using a linear model for the % established in the first column (%test/%training) with the mean value of the BPA at each location. According to this table, the selection of a validation set with 60% of the nodes is enough for the network of measuring platforms. Table 8 shows the results of the cross-correlation test to predict the temporal values of BPA in the outdoor environment and generates a linear model to predict the values of BPA in the test percentage established in the first column (as in the previous case). As in the previous case, we used the whole temporal series in each location and from this results we can predict quite well the test values training the model with the 50% of the measurements (as shown the RMSE and MAE values).

It can be seen that in both cases (the indoor and outdoor environments), the relationship between the BPA and binaural loudness + binaural sharpness is better correlated than the BPA and SPL.

In this case, location 4 in the outdoor environment corresponds to the place of the most exposed node to the traffic noise. Therefore, as the variance of the temporal values for the SPL and the binaural loudness and sharpness is the lowest one (see Table 3), both for the test set and the training set, the random selection of time samples for the LOOCV gave for the RMSE and MAE a very low value in the cases of 80% and 50% of the training set, making a really good matching for the temporal prediction of the SPL and the BPA (from the binaural loudness and sharpness).

### 4.4. Spatial Statistical Analysis (Kriging Method)

Spatial statistics allows the use of several methods. The most common methods are Inverse Distance Weighted (IDW), spline and kriging. IDW is a simple and intuitive deterministic interpolation method based on principle that sample values closer to the prediction location have more influence on prediction value than sample values farther apart. The major disadvantage of IDW is “bull’s eye” effect and edgy surface. Spline is also a deterministic interpolation method which fits mathematical function through input data to create smooth surface. Kriging is a method based on spatial autocorrelation [46].

The computed PA from the measurements of binaural loudness and sharpness establish a data set in relation to different locations with its GPS coordinates, longitude and latitude. By denoting the PA computed with the simplified Zwicker’s model at a location *x* as Y(x), this data set is defined as {Y(x),x∈D}, where D are all the locations of the modelling sets, following the kriging technique [47].

In this context, the objective of this proposed model is the prediction of $Y(x_{0})$ in any location $x_{0}$, particularly those within the validation set. The annoyance reports contain information of the set of covariables included. Therefore, Y(x) is modeled as a tendency function of the covariables better involved in the process which explains its variability in a large extent plus some random error which is explained by the short term variability, i.e.,

(4) Y(x)=μ(x)+δ(x),

where μ(x)=E[Y(x)] and δ(x) is a stationary Gaussian process with zero mean, whose spatial dependence characterization is given by the variogram γ [48]:

(5) $$2\gamma \left(h\right)=\mathrm{Var}\left[Y(x+h)-Y(x)\right]=\mathrm{Var}\left[\delta (x+h)-\delta (x)\right],$$

where Var denotes the variance and *h* is an offset. This variogram represents the main function of the kriging method, which presents different procedures such as simple kriging, ordinary kriging, universal kriging, indicator kriging, co-kriging, etc, attending to different statistical aspects considered in the covariable set. Ordinary kriging is the most widely used kriging method. It is used to estimate a value at a point of a region for which a variogram is known, using data in the neighborhood of the estimation location and also can be used to estimate a block value [49].

**Figure 5:**
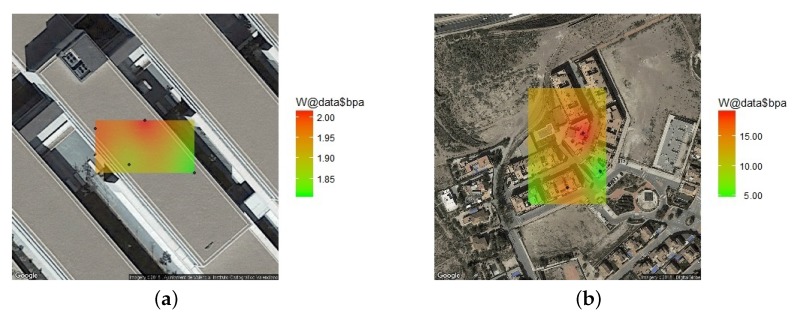
Spatial statistic prediction of BPA (with Spherical Kriging method) for the indoor (**a**) and outdoor environments (**b**).

**Figure 6:**
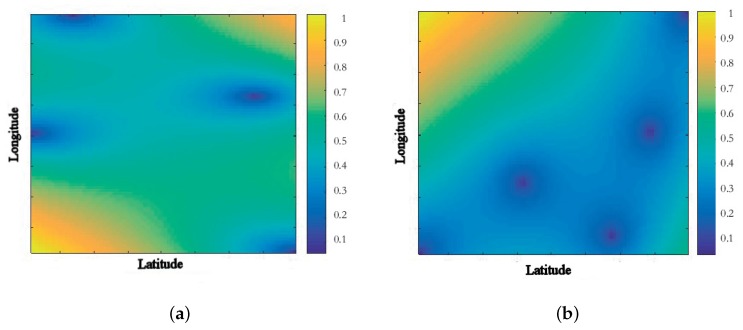
Spatial statistic distribution of relative error from the Kriging method using mean values of BPA in the nodes for the indoor (**a**) and outdoor environments (**b**).

In this study, the variogram has been calculated, using ordinary kriging and a spherical model, with the R statistical package for variogram fitting [50]. Figure 5a shows the spatial distribution of the BPA in the indoor environment and Figure 5b shows the spatial distribution of the BPA in the outdoor environment. The map shows the evaluation of the equation 3 with the kriging method showing the points between the measuring points. Also Figure 6a,b show the relative error of the kriging estimation showing that the error of the Kriging estimation is kept bounded inside the region between the measuring points below 25% and grows up as the estimated point is in the outer part and far away from the centroid of the measurement point.

Figure 6 shows the relative error distribution obtained in the Kriging estimation. The estimation absolute error range for the BPA in the indoor environment (see Figure 6a) is [0.0064, 0.1082] which is reasonably low and is bellow the BPA obtained from measurements. The greater error is focused in the region outside the building where no measurements are done. For the outdoor environment (see Figure 6b), the estimation error range is [0.9525, 24.2659] which is a larger range compared to the BPA obtained from measurements. The greater error is located in the region next to the highway.

### 4.5. A Preliminar Subjective Study

In order to check the subjective response in the indoor and outdoor environments, a short poll was conducted to a group of people related to each environment. The poll asked for some social aspects (gender and age) and the valuation of some aspects related to the soundscape, following the Swedish Soundscape-Quality Protocol which assess the perceived affective quality using the following eight adjectives: Pleasant, Unpleasant, Eventful, Uneventful, Monotonous, Exciting, Calm, and Noisy [51].

An Android application for mobile has been developed with the short poll (translated into Spanish) to allow a quick data gathering of the surveyants. In order to locate the response of every person answering the poll, the GPS coordinates have been registered with the same application.

The analysis of the results of this poll is shown in Table 9.

According to this values the response in the indoor environment is generally qualified as pleasant and mostly uneventful (calm and nearly monotonous), although at certain hours students make the soundscape a bit exciting.

Taking into account the values in the outdoor environment, in general, the values are a little bit higher for noisy and unpleasant, which means that the people living in this area seems to like the place but stand the noise from the highway and like the noise made by children activities.

## 5. Conclusions

In this work, an IoT system collecting information from each acoustic environment in different environments (indoor and outdoor) is deployed. The nodes of the system gather audio information and compute psycho-acoustic parameters (loudness and sharpness) performing the binaural synthesis by considering the HRTFs. In the indoor environment, the nodes were placed in different rooms at the Computer Science Department of the University of Valencia and in the outdoor environment, the nodes were located in the outdoor of different houses in a residential area in Murcia.

From the binaural psycho-acoustic parameters gathered and the SPL, different statistical analysis were done. The correlation between these parameters was performed, showing that the binaural loudness and the SPL correlated well in nearly every location (indoor and outdoor, at a 99% significance level). Also, the binaural sharpness worked fine with the SPL, but with smaller Pearson coefficient values.

From the simplified version of the Zwicker’s Psycho-acoustic Annoyance model, the values of the Binaural Psycho-acoustic Annoyance were computed to determine the subjective annoyance at each node location in the indoor and outdoor environment. At this point, different cross-validation analysis were done in order to determine the validity of the BPA spatial and temporal values. To this end, a Leave-One-Out-Cross-Validation algorithm was used to determine the best modelling set (a good training set was established with 50% of the values). In all cases (indoor and outdoor), the binaural loudness and sharpness explained better the relationship with BPA than the sound pressure level, both for spatial averages and for temporal series. Finally, a spatial statistical analysis was done in the indoor and the outdoor environment by using an Ordinary Kriging technique. The error of the Kriging estimation is kept bounded inside the region between the measuring points below 25% and grows up outside this region. Also a short on-site subjective study has been done. According to the results, the BPA model has been validated for the indoor and outdoor environment. Further work is needed for the spatial validation of the subjective assessment.

Currently, further work is being done with the implementation of the complete Zwicker’s Psycho-acoustic Annoyance model, adding the Roughness and Fluctuation Strength algorithms to the implementation of the binaural model.

## Figures and Tables

**Table 1:** Summary of statistical values (mean and standard deviation) for SPL, binaural loudness and binaural sharpness in the indoor environment.

	Indoor Loc. 1	Indoor Loc. 2	Indoor Loc. 3	Indoor Loc. 4
**SPL(dBA)**	35.61(0.60)	38.98(1.99)	33.75(2.99)	34.13(2.80)
**Binaural Loudness**	1.97(1.01)	1.98(0.39)	1.89(1.44)	1.68(1.36)
**Binaural Sharpness**	1.76(0.10)	1.81(0.05)	1.82(0.15)	2.02(0.13)

**Table 2:** Pearson’s correlation for SPL, binaural loudness and binaural sharpness in the indoor environment.

**Indoor Loc. 1**	**Indoor Loc. 2**
$\begin{array}{cccc} & binld & binshrp & SPL\\ binld & 1.00 & 0.91{\;}^{*} & 0.85{\;}^{**}\\ binshrp & 0.91{\;}^{**} & 1.00 & 0.75{\;}^{**}\\ SPL & 0.85{\;}^{**} & 0.75{\;}^{**} & 1.00 \end{array}$	$\begin{array}{ccc} binld & binshrp & SPL\\ 1.00 & -0.90{\;}^{**} & 0.96{\;}^{**}\\ -0.90{\;}^{**} & 1.00 & -0.86{\;}^{**}\\ 0.96{\;}^{**} & -0.86{\;}^{**} & 1.00 \end{array}$
**Indoor Loc. 3**	**Indoor Loc. 4**
$\begin{array}{cccc} & binld & binshrp & SPL\\ binld & 1.00 & 0.37{\;}^{*} & 0.89{\;}^{**}\\ binshrp & 0.37{\;}^{*} & 1.00 & 0.19\\ SPL & 0.89{\;}^{**} & 0.19 & 1.00 \end{array}$	$\begin{array}{ccc} binld & binshrp & SPL\\ 1.00 & -0.20 & 0.92{\;}^{**}\\ -0.20 & 1.00 & -0.44{\;}^{**}\\ 0.92{\;}^{**} & -0.44{\;}^{**} & 1.00 \end{array}$

${}^{*}$: *p* < 0.05; ${\;}^{**}$: *p* < 0.01; *p* = significance.

**Table 3:** Summary of statistical values (mean and standard deviation) for SPL, binaural loudness and binaural sharpness in the outdoor environment.

	Outdoor Loc. 1	Outdoor Loc. 2	Outdoor Loc. 3	Outdoor Loc. 4	Outdoor Loc. 5
**SPL(dBA)**	38.54(4.84)	50.09(12.45)	39.73(1.93)	51.74(1.24)	53.72(14.02)
**Binaural Loudness**	4.53(2.40)	12.83(14.91)	4.95(0.74)	11.34(0.98)	17.39(15.95)
**Binaural Sharpness**	1.74(0.23)	1.85(0.40)	1.53(0.09)	1.44(0.08)	2.04(0.18)

**Table 4:** Pearson’s correlation for SPL, binaural loudness and binaural sharpness in the outdoor environment.

**Outdoor Loc. 1**	**Outdoor Loc. 2**	**Outdoor Loc. 3**
$\begin{array}{cccc} & binld & binshrp & SPL\\ binld & 1.00 & 0.15 & 0.86{\;}^{**}\\ binshrp & 0.15 & 1.00 & 0.40{\;}^{**}\\ SPL & 0.86{\;}^{**} & 0.40{\;}^{**} & 1.00 \end{array}$	$\begin{array}{ccc} binld & binshrp & SPL\\ 1.00 & 0.87{\;}^{**} & 0.97{\;}^{**}\\ 0.87{\;}^{**} & 1.00 & 0.89{\;}^{**}\\ 0.97{\;}^{**} & 0.89{\;}^{**} & 1.00 \end{array}$	$\begin{array}{ccc} binld & binshrp & SPL\\ 1.00 & 0.63{\;}^{**} & 0.96{\;}^{**}\\ 0.63{\;}^{**} & 1.00 & 0.58{\;}^{**}\\ 0.96{\;}^{**} & 0.58{\;}^{**} & 1.00 \end{array}$
**Outdoor Loc. 4**	**Outdoor Loc. 5**	
$\begin{array}{cccc} & binld & binshrp & SPL\\ binld & 1.00 & 0.26{\;}^{**} & 0.21\\ binshrp & 0.26{\;}^{**} & 1.00 & 0.96{\;}^{**}\\ SPL & 0.21 & 0.96{\;}^{**} & 1.00 \end{array}$	$\begin{array}{ccc} binld & binshrp & SPL\\ 1.00 & 0.30 & 0.88{\;}^{**}\\ 0.30 & 1.00 & -0.02\\ 0.88{\;}^{**} & -0.02 & 1.00 \end{array}$	

${}^{*}$: *p* < 0.05; ${\;}^{**}$: *p* < 0.01; *p* = significance.

**Table 5:** Summary of spatial average Leave-One-Out-Cross-Validation test with a Linear Model method for the indoor environment.

	BPA vs. BinLdn+BinShrp			BPA vs. BinSPL		
Test%/Training%	RMSE	Rsquared	MAE	RMSE	Rsquared	MAE
25%/75%	0.1642	0.6842	0.1095	0.0809	0.1896	0.0745
50%/50%	0.02593687	1	0.02593687	0.02593687	1	0.02593687

**Table 6:** Summary of temporal Leave-One-Out-Cross-Validation test with a Linear Model method in each location for the indoor environment.

		BPA vs. BinLdn+BinShrp			BPA vs. BinSPL		
	Test%/Training%	RMSE	Rsquared	MAE	RMSE	Rsquared	MAE
Location 1	10%/90%	0.0605	0.9993	0.0112	0.5316	0.0069	0.2323
	20%/80%	0.0648	0.9995	0.0121	0.5658	0.0069	0.2379
	30%/70%	0.0002	0.9999	0.0002	0.0429	0.0539	0.0333
	40%/60%	0.0002	0.9999	0.0002	0.0434	1.707e-05	0.0345
	50%/50%	0.0002	0.9999	0.0002	0.0455	0.0314	0.0366
Location 2	10%/90%	0.0061	0.9999	0.0047	0.2725	0.9206	0.2090
	20%/80%	0.0058	0.9999	0.0045	0.2676	0.9408	0.2075
	30%/70%	0.0055	0.9999	0.0040	0.2702	0.9160	0.2094
	40%/60%	0.0063	0.9999	0.0049	0.2808	0.9256	0.2231
	50%/50%	0.0081	0.9998	0.0056	0.3702	0.6241	0.2407
Location 3	10%/90%	0.0774	0.9978	0.0375	0.8210	0.7546	0.5782
	20%/80%	0.0907	0.9957	0.0395	0.7292	0.7217	0.5071
	30%/70%	0.0817	0.9979	0.0356	0.9079	0.7355	0.6545
	40%/60%	0.0473	0.9993	0.0229	0.9071	0.7418	0.6455
	50%/50%	0.0869	0.9874	0.0370	0.6180	0.3623	0.3723
Location 4	10%/90%	0.0239	0.9998	0.0142	0.8508	0.6841	0.4118
	20%/80%	0.0225	0.9988	0.0138	0.3474	0.7180	0.2322
	30%/70%	0.0241	0.9982	0.0142	0.3714	0.5553	0.2362
	40%/60%	0.0281	0.9998	0.0165	1.1018	0.6026	0.5194
	50%/50%	0.0291	0.9998	0.0137	0.7437	0.8487	0.4502

**Table 7:** Summary of spatial average Leave-One-Out-Cross-Validation test with a Linear Model method for the outdoor environment.

	BPA vs. BinLdn+BinShrp			BPA vs. BinSPL		
Test%/Training%	RMSE	Rsquared	MAE	RMSE	Rsquared	MAE
20%/80%	1.9719	0.8255	1.8525	4.1603	0.4163	3.1738
40%/60%	3.0859	0.9893	1.9617	4.8855	0.7218	3.3964

**Table 8:** Summary of temporal Leave-One-Out-Cross-Validation test with a Linear Model method in each location for the outdoor environment.

		BPA vs. BinLdn+BinShrp			BPA vs. BinSPL		
	Test%/Training%	RMSE	Rsquared	MAE	RMSE	Rsquared	MAE
Location 1	10%/90%	0.1486	0.9966	0.1017	1.4697	0.6646	0.8459
	20%/80%	0.1053	0.9984	0.0786	1.5376	0.6491	0.8508
	30%/70%	0.1633	0.9964	0.1090	1.6677	0.6202	0.9639
	40%/60%	0.1671	0.9886	0.1192	0.8162	0.7285	0.5695
	50%/50%	0.1115	0.9987	0.0824	1.9067	0.6212	1.1335
Location 2	10%/90%	1.9266	0.9927	1.2462	7.2511	0.8973	5.3961
	20%/80%	1.9589	0.9939	1.2616	7.8270	0.9025	5.7730
	30%/70%	2.0966	0.9933	1.3753	7.6228	0.9119	5.9002
	40%/60%	1.7522	0.9918	1.2066	6.8213	0.8759	5.1862
	50%/50%	2.4489	0.9849	1.4635	8.6762	0.8098	5.8777
Location 3	10%/90%	0.0392	0.9960	0.0209	0.2068	0.8892	0.1478
	20%/80%	0.0460	0.9969	0.0287	0.2445	0.9109	0.1722
	30%/70%	0.0456	0.9971	0.0298	0.2753	0.8931	0.1912
	40%/60%	0.0516	0.9965	0.0291	0.2873	0.8868	0.2036
	50%/50%	0.0429	0.9979	0.0280	0.2988	0.8924	0.2026
Location 4	10%/90%	0.0945	0.9914	0.0367	0.5353	0.7214	0.3166
	20%/80%	1.175e-15	1	7.772e-16	0.3548	0.8542	0.2496
	30%/70%	0.1060	0.9907	0.0442	0.5217	0.7693	0.2925
	40%/60%	0.1180	0.9855	0.0492	0.5771	0.6487	0.3158
	50%/50%	7.390e-16	1	3.075e-16	0.4369	0.7464	0.2995
Location 5	10%/90%	0.8829	0.9975	0.7682	13.889	0.3949	9.1463
	40%/60%	0.9831	0.9974	0.7740	16.168	0.2923	11.448
	50%/50%	0.6828	0.9990	0.6348	13.927	0.5045	10.464

**Table 9:** Summary of statistical values for the evaluation of the on-site subjective survey.

	Indoor	Outdoor
% Gender (male/female)	40/60	46/54
% Ages (18-30, 30-45, 45-55, 55-65)	20/65/15/0	8/69/23/0
Mean and standard deviation values of:		
Pleasant	4.35/0.45	3.96/0.71
Unpleasant	1.65/0.45	2.10/0.58
Eventful	2.14/0.52	2.11/0.86
Uneventful	3.96/0.65	4.08/0.86
Exciting	2.38/0.47	1.67/0.65
Monotonous	4.36/0.45	3.84/0.80
Calm	4.65/0.35	4.08/0.76
Noisy	1.93/0.85	2.23/1.09

## Abbreviations

The following abbreviations are used in this manuscript:

BLE	Bluetooth Low Energy
(B)PA	(Binaural) Psycho-acoustic Annoyance
DOA	Direction of Arrival
HRTF	Head Related Transfer Function
IoT	Internet of Things
ITS	Intelligent Transportation Systems
LM	Linear Model
LOOCV	Leave-One-Out-Cross-Validation
MAE	Maximum Absolute Error
RMSE	Root Mean Squared Error
SPL	Sound Pressure Level
WASN	Wireless Acoustic Sensor Network
